# A Novel Platform for Research on Toxins

**DOI:** 10.3390/toxins1010001

**Published:** 2009-07-06

**Authors:** Florian Lang

**Affiliations:** Physiologisches Institut I, Universität Tübingen, Gmelinstrasse 5, D-72076 Tübingen, Germany; Email: florian.lang@uni-tuebingen.de; Tel.: +49 7071 29 72194; Fax: +49 7071 29 5618

Toxins are ubiquitous in nature and as such they impact our daily life. Toxins may come from a wide variety of sources and influence a myriad of biological functions. Research on toxins may address their production, structure, chemical properties, biological activity, and economic impact. On the one hand, toxins may be used to decipher biological mechanisms, to favourably influence disease or to combat unwanted organisms. On the other hand, toxins may be a hazard jeopardizing health of humans, animals or plants.

In view of the many aspects of research on toxins, it is not surprising that it attracts scientists from a wide variety of scientific disciplines. The significance of toxin-related research is illustrated by the more than two hundred thousand papers dealing with some aspect of toxins. The authors of those papers are from a large number of chemical and biomedical disciplines including toxicology, pharmacology, molecular biology, biochemistry, physiology, pathophysiology and pathobiochemistry from molecules to integrated function of organisms. Progress in understanding the properties and effects of toxins critically depends on the mutual interaction of scientists from these different disciplines. 

The journal *Toxins* aims to foster the crosstalk between scientists of different methodological and theoretical backgrounds, addressing the various aspects of toxin chemistry and toxin action. The scope of this journal is probably best reflected by the research interests of the members in the editorial board. However, the journal will publish any manuscript of high scientific quality which pertains to toxin chemistry and toxin effect irrespective of the questions asked, the methods applied or the material analyzed. By competent, rapid and fair reviewing the journal attempts to attract authors and by publishing excellent papers to attract readers.

The open access of the journal will provide maximal dissemination of the published research reaching an almost unlimited readership. In this way, the large and heterogeneous scientific community interested in toxins will be reached without any restrictions. Accordingly, the access will allow the journal to optimally serve its function, i.e., the promotion of research by distributing pertinent experimental knowledge and scientific ideas.

